# BanglaOCT2025: A Population-Specific Fovea-Centric OCT Dataset with Self-Supervised Volumetric Restoration Using Flip-Flop Swin Transformers

**DOI:** 10.3390/diagnostics16030420

**Published:** 2026-02-01

**Authors:** Chinmay Bepery, G. M. Atiqur Rahaman, Rameswar Debnath, Sajib Saha, Md. Shafiqul Islam, Md. Emranul Islam Abir, Sanjay Kumar Sarker

**Affiliations:** 1 Department of Computer Science and Information Technology, Patuakhali Science and Technology University, Patuakhali 8602, Bangladesh; 2 Computer Science and Engineering Discipline, Khulna University, Khulna 9208, Bangladesh; rdebnath@cse.ku.ac.bd; 3 The Australian e-Health Research Centre, CSIRO, Perth, WA 6151, Australia; sajib.saha@csiro.au; 4 Department of Ophthalmology, Sher-e-Bangla Medical College Hospital, Barishal 8200, Bangladesh; shafiq_islam326@yahoo.com; 5 Department of Ophthalmology, Khulna Medical College Hospital, Khulna 9100, Bangladesh; emran2590@gmail.com; 6 Department of Vitreo-Retina, National Institute of Ophthalmology and Hospital, Dhaka 1207, Bangladesh; twisansarker@gmail.com

**Keywords:** BanglaOCT2025, Optical Coherence Tomography, fovea detection, speckle noise reduction, self-supervised learning, Flip-Flop Swin Transformer, AMD

## Abstract

**Background:** Age-related macular degeneration (AMD) is a major cause of vision loss, yet publicly available Optical Coherence Tomography (OCT) datasets lack demographic diversity, particularly from South Asian populations. Existing datasets largely represent Western cohorts, limiting AI generalizability. Moreover, raw OCT volumes contain redundant spatial information and speckle noise, hindering efficient analysis. **Methods:** We introduce BanglaOCT2025, a retrospective dataset collected from the National Institute of Ophthalmology and Hospital (NIOH), Bangladesh, using Nidek RS-330 Duo 2 and RS-3000 Advance systems. We propose a novel preprocessing pipeline comprising two stages: (1) A constraint-based centroid minimization algorithm automatically localizes the foveal center and extracts a fixed 33-slice macular sub-volume, robust to retinal tilt and acquisition variability; and (2) A self-supervised volumetric denoising module based on a Flip-Flop Swin Transformer (FFSwin) backbone suppresses speckle noise without requiring paired clean reference data. **Results:** The dataset comprises 1585 OCT volumes (202,880 B-scans), including 857 expert-annotated cases (54 DryAMD, 61 WetAMD, and 742 NonAMD). Denoising quality was evaluated using reference-free volumetric metrics, paired statistical analysis, and blinded clinical review by a retinal specialist, confirming preservation of pathological biomarkers and absence of hallucination. Under a controlled paired evaluation using the same classifier with frozen weights, downstream AMD classification accuracy improved from 69.08% to 99.88%, interpreted as an upper-bound estimate of diagnostic signal recoverability rather than independent generalization. **Conclusions:** BanglaOCT2025 is the first clinically validated OCT dataset representing the Bengali population and establishes a reproducible fovea-centric volumetric preprocessing and restoration framework for AMD analysis, with future validation across independent and multi-centre test cohorts.

## 1. Introduction

Optical Coherence Tomography (OCT) functions as an “optical ultrasound,” utilizing low-coherence interferometry to provide non-invasive, micrometer-scale cross-sectional views of retinal microstructures [1]. In these grayscale tomograms, pixel intensity correlates with tissue backscattering properties; hyper-reflective (bright) regions indicate distinct layers such as the Retinal Nerve Fiber Layer (RNFL) and the Retinal Pigment Epithelium (RPE), while hypo-reflective (dark) regions represent the nuclear layers and vitreous humor [2]. Even minor textural or intensity deviations within these layers can signal early pathological changes associated with sight-threatening conditions.

OCT has become the clinical gold standard for diagnosing age-related macular degeneration (AMD) and other macular disorders [3]. However, the development of reliable automated diagnostic systems is constrained by two fundamental challenges: demographic dataset bias and volumetric image quality degradation.

First, majority of the publicly available OCT datasets, such as Duke SD-OCT, OCTA-500, and AROI, are based on Western (largely Caucasian) or East Asian patient cohorts [4,5,6]. Although these datasets are well curated and widely used, they provide limited representation of South Asian populations, where retinal anatomy, pigmentation, and disease characteristics may differ across ethnic groups [7]. As a result, AI models trained on Western OCT datasets may experience domain shift when applied to South Asian populations, reducing diagnostic reliability. Despite the growing burden of age-related macular degeneration, population-specific OCT datasets for South Asia—particularly Bangladesh—remain scarce. Such anatomical and demographic differences can alter OCT appearance and challenge model generalization, consistent with broader findings on limited cross-population transferability in medical AI [8].

To address this gap, we introduce **BanglaOCT2025**, a large-scale macular OCT dataset collected at the National Institute of Ophthalmology and Hospital (NIOH), Bangladesh, using NIDEK RS-330 Duo 2 and RS-3000 Advance systems under routine clinical workflows, with clinician-verified annotations and demographic metadata [9,10].

Second, the volumetric nature of OCT introduces redundancy and speckle noise. Standard macular scans contain up to 128 B-scans covering a 6 mm × 6 mm retinal region, many of which lie outside the diagnostically relevant central macula and increase computational cost and noise sensitivity [11]. Reliable 3D analysis, therefore, requires accurate localization of the fovea centralis; however, existing fovea detection methods based on global thickness or intensity heuristics are often sensitive to tilt, motion artifacts, and pathological deformation [12,13,14].

To address this, we propose a constraint-based centroid minimization algorithm that robustly localizes the fovea, even in tilted scans, and extracts a standardized 33-slice sub-volume. This fovea-centric design aligns with clinical practice, where AMD-related biomarkers are concentrated in the central macula, while reducing redundant computation.

Furthermore, raw OCT images are inherently affected by speckle noise [15]; it appears as a granular interference pattern that obscures fine pathological features, including drusen boundaries, subretinal fluid, and outer retinal layers. Conventional two-dimensional filtering methods often reduce this noise at the cost of blurring layer boundaries, thereby compromising diagnostic detail [16,17]. Recent machine-learning approaches, mainly based on 2D autoencoders, denoise OCT images by processing individual B-scans and typically rely on paired or reference images [18,19,20,21]. However, such methods largely ignore the intra- and inter-slice spatial relationships inherent to volumetric OCT data. Consequently, the use of collocated volumetric coherence for self-supervised OCT denoising remains underexplored.

To address this limitation, we introduce a self-supervised volumetric restoration framework based on a Flip-Flop Swin Transformer (FFSwin) backbone. By leveraging intra- and inter-slice anatomical coherence, the model suppresses speckle noise while preserving retinal structure, without requiring noise-free reference images.

In summary, this study makes four main contributions: (i) the introduction of BanglaOCT2025, the first clinically curated macular OCT dataset representing the Bengali population; (ii) a tilt-robust, constraint-based method for automated fovea-centric sub-volume extraction; (iii) a self-supervised volumetric denoising framework using an FFSwin backbone; and (iv) a comprehensive evaluation demonstrating that restoration-driven preprocessing enhances downstream AMD classification under severe class imbalance. Downstream classification is used solely as a task-oriented probe to assess preservation of diagnostically relevant information, while the primary focus of this work remains the dataset, preprocessing pipeline, and volumetric restoration methodology.

## 2. Materials and Methods

### 2.1. Dataset: BanglaOCT2025

#### 2.1.1. Dataset Acquisition

BanglaOCT2025 was retrospectively collected (BanglaOCT2025) from the National Institute of Ophthalmology and Hospital (NIOH), Bangladesh, under routine clinical workflows. All data were de-identified in accordance with institutional data governance practices prior to analysis, with only limited demographic metadata (age and sex) retained for research purposes. No personally identifiable information was used in this study.

OCT examinations were acquired using NIDEK spectral-domain OCT systems, namely the RS-330 Duo 2 and RS-3000 Advance (NIDEK Co., Ltd., Gamagori, Japan), operated through the NAVIS-EX image management software. A total of 1419 patient records were initially retrieved from the NAVIS-EX system, comprising 1071 cases from the RS-330 Duo 2 and 348 cases from the RS-3000 Advance. After excluding incomplete, corrupted, or empty scan folders, 1071 valid patients and 1658 volumetric OCT scans were retained. A detailed breakdown of patient counts, scan validity, and slice statistics is summarized in Table 1.

Each macular OCT volume was acquired using a standard 6 mm × 6 mm fovea-centered protocol with 128 B-scans, following routine clinical practice including patient fixation guidance, optional pupil dilation, automated alignment, and manual confirmation of macular center to ensure consistent alignment and image quality for AMD assessment [2,23,24,25,26]. All B-scans were stored as grayscale images, where pixel intensity represents tissue backscattering and supports layer-wise (e.g., RNFL and RPE) retinal analysis.

Following quality control, a total of 1585 scans (202,880 B-scans) were retained for the BanglaOCT2025 dataset, as summarized in Table 2. Among these, 857 scans from 573 patients were selected for expert annotation. Diagnostic labels were assigned by experienced retina specialists into three clinical categories: DryAMD, WetAMD, and NonAMD. Each case was independently reviewed by multiple clinicians, and final labels were determined through consensus to enhance annotation reliability. This expert-driven labeling strategy is consistent with established best practices in ophthalmic imaging datasets [3,4,27]. Scans without expert labels were retained for unsupervised and restoration-only experiments.

To our knowledge, BanglaOCT2025 is the first large-scale, clinically curated OCT dataset focused on the Bengali population. By offering population-specific data with verified clinical labels, it fills an important gap in existing OCT resources and supports the development of robust ophthalmic AI systems for South Asian populations.

#### 2.1.2. Ground Truth Labeling

To ensure clinical reliability, the dataset underwent a rigorous labeling process involving three retina specialists from Sher-e-Bangla Medical College Hospital (SBMCH), Khulna Medical College Hospital (KMCH), and NIOH. The specialists independently reviewed scans using the Nidek NAVIS-EX software (trial)version-1.12 19702-E201.

Two specialists (SBMCH and KMCH) independently labeled 573 patients (857 scans) as DryAMD, WetAMD, or NonAMD. Most cases showed consistent agreement; diagnostically ambiguous cases—primarily early-stage distinctions between DryAMD and WetAMD—were reviewed by a third specialist from NIOH. The final ground-truth labels were determined by a majority consensus, reflecting standard clinical practice [27,28]. The annotated dataset includes 857 scans (54 DryAMD, 61 WetAMD, and 742 NonAMD). The NonAMD category comprises both normal eyes and non-AMD retinal conditions, consistent with a clinically realistic “AMD vs. non-AMD” screening formulation.

Inter-grader reliability was assessed prior to consensus labeling. Agreement between the two primary graders across all 857 cases yielded a Cohen’s κ of 0.78, indicating substantial agreement. Of these, 812 cases were labeled identically, while 45 challenging cases were adjudicated by the third grader. Fleiss’ κ computed on this adjudicated subset was low (κ ≈ 0.01), reflecting the intentionally difficult nature of these borderline cases rather than poor annotation quality.

The BanglaOCT2025 dataset includes 1585 scans, including 728 unlabeled scans from 1071 patients aged 10.5–85.5 years. Ground-truth labels were assigned to patients aged 48.5–85.5 years, with the youngest WetAMD case observed at 49.5 years. In ground-truth labelling, a total of 81 patients are considered, ranging in age from 46 to 50.5 years, among 141 patients. Patient distribution according to age is summarized in Table 3. In this dataset, gender-wise dry AMD and wet AMD are presented in Table 4.

### 2.2. Constraint-Based Fovea-Centric Volume Extraction

Each B-scan was exported as an 8-bit grayscale bitmap image $I^{\left(i\right)}\;\in \;{\mathbb{R}}_{\left[0,255\right]}^{\left(H\times W\right)},\;$ where i=1, 2,…,128 denotes the slice index, H and W represent the image height and width, respectively.

Accurate localization of the fovea is a critical prerequisite for reliable macular analysis, as automated fovea detection may be unreliable in the presence of retinal pathology, fixation instability, or scan tilt. Prior studies have shown that pathological deformation and acquisition artifacts can significantly affect automated fovea localization accuracy [12,13]. Unlike prior fovea detection approaches that rely on global thickness profiles or intensity heuristics, the proposed method introduces a constraint-aware, column-wise centroid formulation that is robust to tilt and pathological deformation. Therefore, for algorithm validation and benchmarking purposes only, the foveal center was manually identified following established clinical protocols reported in the literature [12,13]. All preprocessing and downstream experiments in this study rely exclusively on the proposed fully automated constraint-based centroid minimization algorithm, without manual intervention. During validation only, manual foveal localization was performed by identifying the B-scan with the deepest foveal pit within ±5 slices of the system-reported fovea, following established clinical protocols [29]. After foveal localization, a standardized fovea-centered sub-volume of 33 consecutive B-scans (fovea ±16 slices) was extracted to ensure consistent macular coverage across all subjects [11]. To reduce computational cost, a fovea-centered sub-volume of 33 slices was used. Although the fovea is typically located near the mid-volume (around slice 64), anatomical variation and patient motion can shift its position between slices 59 and 69 [12]. To ensure robust detection, the algorithm segments retinal tissue in the central region of each slice, computes a column-wise centroid to identify the retinal pit, applies a slice-level pit metric with anatomical penalty constraints, and extracts the foveal slice along with its 16 neighboring slices on each side.

In summary, the proposed constraint-based centroid minimization framework enables reliable, tilt-robust automated foveal localization and standardized 33-slice macular sub-volume extraction (Implementation Link). Algorithm 1 summarizes the proposed constraint-based fovea detection pipeline. Manual foveal identification is used solely for validation, while all reported analyses are based on the fully automated pipeline.
**Algorithm**
**1**:  Constraint-Based Centroid Minimization Parameters:
•
$N_{\mathrm{adj}}=16$ (Adjacent slices on each side)•
$T_{\mathrm{slices}}=128$ (Total slices per volume)•
$R_{\mathrm{start}}=59$ $,\;R_{\mathrm{end}}=69$ (preferred foveal range)•
P=200 (Penalty for out-of-range slices) Procedure: For each patient folder 
F
:
a.
Initialize metric dictionary M←{}
b.For each slice i=1  to 128 :

Load image I←oct_c_i.bmp

$\mathrm{Extract}\;\mathrm{central}\;\mathrm{region}\;(35\%\;\mathrm{width}):\;I_{c}\leftarrow I[:,0.325W:0.675W]$

$\mathrm{Apply}\;\mathrm{Gaussian}\;\mathrm{blur}:\;I_{b}\leftarrow \mathrm{GaussianBlur}(I_{c},(7,7))$

$\mathrm{Compute}\;\mathrm{Otsu}\;\mathrm{threshold}\;T_{\mathrm{Otsu}}$

$\mathrm{Segment}\;\mathrm{tissue}:\;B\leftarrow (I_{b}\geq T_{\mathrm{Otsu}})$

$\mathrm{Compute}\;\mathrm{column}\;\mathrm{centroids}\;c_{y}$

$M_{\mathrm{pit}}\leftarrow min(c_{y})$

If i<59  or i>69 $:\;M[i]\leftarrow M_{\mathrm{pit}}+200$

$\mathrm{Else}:\;M[i]\leftarrow M_{\mathrm{pit}}$
c.
$\mathrm{Find}\;\mathrm{foveal}\;\mathrm{slice}:\;i_{\mathrm{fovea}}\leftarrow argmin(M)$
d.
$\mathrm{Calculate}\;\mathrm{range}:\;k_{\mathrm{start}}\leftarrow max(1,i_{\mathrm{fovea}}-16)$ $,\;k_{\mathrm{end}}\leftarrow min(128,i_{\mathrm{fovea}}+16)$
e.
$\mathrm{If}\;k_{\mathrm{end}}>128$ : adjust range leftwardf.
$\mathrm{If}\;k_{\mathrm{start}}<1$ : adjust range rightwardg.
$\mathrm{Copy}\;\mathrm{slices}\;k_{\mathrm{start}}$ $\;\mathrm{through}\;k_{\mathrm{end}}$ to the output folder

#### 2.2.1. Tilt-Robust Foveal Slice Detection and Macular Extraction Algorithm

A novel fovea detection algorithm was employed that integrates column-wise centroid analysis [30] with clinical range constraints, enabling robust slice localization under image rotation and tilt.

***Central Region Extraction:*** To reduce peripheral noise while preserving the foveal pit, analysis was restricted to the central macular region, consistent with prior macula-focused OCT studies [11,31]. For anatomical relevance and computational efficiency, a central region covering 35% of the image width was extracted. This width was chosen to reliably include the foveal and parafoveal regions while excluding peripheral areas more prone to shadowing and curvature artifacts.

The parameters used for fovea detection (penalty value = 200, central width = 35%, and foveal slice range = 59–69) were chosen based on known macular anatomy and typical OCT acquisition geometry rather than dataset-specific fine-tuning. These settings define broad spatial constraints using relative proportions and slice indices, rather than absolute intensity thresholds. During method development, we observed that moderate parameter variations (e.g., ±5–8% change in central width or small shifts in slice range) did not meaningfully affect fovea localization, indicating that the procedure is not overly sensitive to precise parameter settings. Nevertheless, atypical scanning protocols may require adjustment, and systematic cross-scanner sensitivity analysis is left for future work.

The central region extraction is defined as:

(1) $$I_{\mathrm{center}}^{\left(i\right)}(x,y)\;=\;I^{\left(i\right)}\left(x,y_{\mathrm{start}}^{\left(i\right)}:y_{\mathrm{end}}^{\left(i\right)}\right)$$

where, $y_{\mathrm{start}}^{\left(i\right)}\;=\;\left\lfloor W\times 0.325\right\rfloor \;=\;\left\lfloor W\times (0.5-0.175)\right\rfloor$ and $y_{\mathrm{end}}^{\left(i\right)}\;=\;\left\lfloor W\times 0.675\right\rfloor \;=\;\left\lfloor W\times (0.5\;+\;0.175)\right\rfloor$ .

***Denoising with Gaussian Filtering***: Speckle noise [15] was reduced using a 2D Gaussian filter with kernel size 7×7 . A mild Gaussian smoothing σ≈1.0 was applied to suppress speckle-induced high-frequency fluctuations without blurring retinal layer boundaries while preserving layer geometry, consistent with prior OCT preprocessing studies [32,33].

(2) $$I_{\mathrm{blur}}^{\left(i\right)}\;=\;I_{\mathrm{center}}^{\left(i\right)}\times G$$

where $G(x,y)\;=\;\frac{1}{2\pi \sigma^{2}}exp\left(-\frac{x^{2}\;+\;y^{2}}{2\sigma^{2}}\right),\;\sigma \;=\;\frac{7\;-1}{6}\approx 1.0$ .

***Adaptive Thresholding with Otsu’s Method***: Retinal tissue was segmented using Otsu’s method, which automatically determines the optimal threshold $T_{\mathrm{Otsu}}$ by maximizing between class variance to separate retinal tissue from background.

(3) $$B^{\left(i\right)}(x,y)\;=\;\left\{\begin{array}{ll} 1 & \mathrm{if}\;I_{\mathrm{blur}}^{\left(i\right)}(x,y)\geq T_{\mathrm{Otsu}}\\ 0 & \mathrm{otherwise} \end{array}\right.$$

where $T_{\mathrm{Otsu}}$ is computed as:

$$T_{\mathrm{Otsu}}\;=\;arg\underset{0\leq k<256}{max}\left[\omega_{0}(k)\omega_{1}(k){\left(\mu_{0}(k)-\mu_{1}(k)\right)}^{2}\right]$$

This procedure automatically finds the best intensity value to separate bright retinal tissue from darker background by maximizing the difference between the averages of the two groups of pixels.

***Column-Wise (A-Scan) Centroid Analysis:*** The key innovation is computing centroids for each vertical column (A-scan) independently, rather than a single global centroid. This provides tilt-robustness. Unlike global centroid or thickness-based approaches, column-wise centroid estimation preserves robustness under scan tilt and local deformation [12,31]. For each segmented slice $B^{\left(i\right)}$ , the j -th A-scan is represented by a binary depth profile $b_{j}(x)\;=\;B^{\left(i\right)}(x,j),x\;=\;1,2,\ldots ,H,$ capturing axial retinal tissue distribution. The zeroth moment (area) for the column j is $M_{00}^{\left(i,j\right)}\;=\;\sum_{x=1}^{H}B^{\left(i\right)}(x,j)$ , and the first vertical moment is $M_{01}^{\left(i,j\right)}\;=\;\sum_{x=1}^{H}x\cdot B^{\left(i\right)}(x,j)$ .

The column centroid (vertical position) is:

(4) $$C_{y}^{\left(i,j\right)}=\left\{\begin{array}{ll} \frac{M_{01}^{\left(i,j\right)}}{M_{00}^{\left(i,j\right)}} & \mathrm{if}\;M_{00}^{\left(i,j\right)}>\epsilon \\ H & \mathrm{otherwise}\;(\mathrm{no}\;\mathrm{tissue}) \end{array}\right.$$

where $\epsilon =10^{-6}$ prevents division by zero.

***Foveal Pit Metric—Minimum Column Centroid:*** The foveal pit corresponds to the thinnest point of the retina, which manifests as the highest position in the image (minimum y -coordinate). The metric for the slice i is:

(5) $$M_{\mathrm{pit}}^{\left(i\right)}\;=\;\underset{j\in J}{min}C_{y}^{\left(i,j\right)}$$

where J is the set of all columns in the central region. This metric identifies the minimum (highest) centroid among all columns, corresponding to the foveal depression.

***Efficient Vectorized Implementation:*** The algorithm uses NumPy vectorization for computational efficiency. Let $B\in R^{H\times W_{c}}$ denote the segmented central retinal region, where $W_{c}=y_{\mathrm{end}}-y_{\mathrm{start}}$ . The column-wise zeroth and first-order moments are computed as $m_{00}=B^{⊤}1,\;m_{01}=B^{⊤}y$ **,** where 1 is a vector of ones and $y=[1,\ldots ,H{]}^{⊤}$ . The column centroids are then defined as:

(6) $$c_{y}=\left\{\begin{array}{ll} \frac{m_{01}}{m_{00}}, & m_{00}>\epsilon \\ H, & \mathrm{otherwise} \end{array}\right.$$

with $\epsilon =10^{-6}$ to avoid division by zero. Finally,

(7) $$M_{\mathrm{pit}}^{\left(i\right)}=min(c_{y})$$

***Clinical Range Constraint with Penalty Function:*** To incorporate anatomical prior knowledge, slices outside the clinically expected foveal range (59–69) were penalized:

(8) $${\tilde{M}}_{\mathrm{pit}}^{\left(i\right)}\;=\;\left\{\begin{array}{ll} M_{\mathrm{pit}}^{\left(i\right)} & \mathrm{if}\;59\;\leq \;i\;\leq \;69\\ M_{\mathrm{pit}}^{\left(i\right)}\;+\;200 & \mathrm{otherwise} \end{array}\right.$$

A penalty value of 200 was empirically chosen to suppress anatomically implausible slices while retaining strong foveal pit responses, ensuring a balance between robustness and flexibility. These values were chosen to represent anatomically plausible ranges rather than optimized thresholds, and were held fixed across all experiments.

***Foveal Slice Identification:*** The optimal foveal slice index is:

(9) $$i_{\mathrm{fovea}}\;=\;\underset{i\in \{1,2,\ldots ,128\}}{argmin}\;{\tilde{M}}_{\mathrm{pit}}^{\left(i\right)}$$

***Macular Sub-volume Extraction:*** A standardized macular sub-volume centered on the detected fovea was extracted:

(10) $$V_{\mathrm{macula}}\;=\;\left\{I^{\left(k\right)}:k_{\mathrm{start}}\leq k\leq k_{\mathrm{end}}\right\}$$

where $k_{\mathrm{start}}\;=\;max\left(1,i_{\mathrm{fovea}}-16\right)\;and{\;\;k}_{\mathrm{end}}\;=\;min(128,i_{\mathrm{fovea}}\;+\;16)$ .

A fixed 33-slice macular sub-volume centered on the detected fovea was extracted. Boundary conditions were handled by shifting the extraction window to ensure a consistent slice count without exceeding volume limits. Although rare outliers were observed in severely distorted or off-center scans, boundary-aware correction ensured anatomically valid sub-volume extraction in all cases. 

#### 2.2.2. Design Rationale and Robustness Analysis

***Tilt-Robustness Analysis:*** Traditional global centroid methods fail when OCT scans are tilted. Consider a tilted scan where the retinal surface forms an angle θ with the horizontal. The true foveal pit depth $\Delta y_{\mathrm{true}}$ is preserved in column-wise minima but lost in global averaging:

(11) $$C_{y}^{\mathrm{global}}=\frac{1}{W_{c}}\sum_{j}C_{y}^{\left(j\right)}\approx \bar{y}+\frac{\Delta y_{\mathrm{true}}}{2}\cdot tan\theta$$

The method avoids this bias by computing ${min}_{j}C_{y}^{\left(j\right)}$ , which remains invariant to tilt.

***Penalty Function***: The penalty value P=200 was chosen such that:

$$P>\underset{i\in [59,69]}{max}M_{\mathrm{pit}}^{\left(i\right)}-\underset{i\notin [59,69]}{min}M_{\mathrm{pit}}^{\left(i\right)}$$

This ensures the preferred range dominates unless an out-of-range slice has an exceptionally strong foveal signal.

***Computational Complexity:*** The algorithm operates in $O(HW_{c})$ per slice, with vectorized operations achieving near-optimal performance. The total processing time for N patients is approximately $O(128NHW_{c})$ . On a standard workstation (12th Generation Intel® Core™ i7-1255U Processor, Santa Clara, CA, USA), fovea detection required <0.3 s per volume, enabling scalable preprocessing of large datasets.

#### 2.2.3. Parameter Summary

This subsection summarizes the key algorithmic parameters and anatomically motivated constraints used for tilt-robust foveal localization. These settings were selected based on clinical priors and empirical validation on the BanglaOCT2025 dataset. The complete parameter configuration and corresponding rationale are provided in Table 5.

**Table 5:** Algorithm parameters for tilt-robust foveal detection.

Parameters	Value and Rationale
$\mathrm{Total}\;\mathrm{slices}\;(T_{\mathrm{slices}}$ )	128 (standard NIDEK protocol)
$\mathrm{Adjacent}\;\mathrm{slices}\;(N_{\mathrm{adj}}$ )	16 + fovea + 16 ⇒ 33-slice sub-volume covering ∼ 1.5 mm
Search range	59–69 (clinically expected foveal location 64 ± 5 slices)
Penalty value (P )	200 (empirically validated on the BanglaOCT2025 dataset)
Central width	35% of the image (focus on the anatomically relevant region)
Gaussian kernel	7×7 (σ≈1.0 , balances noise reduction and edge preservation)
Threshold method	Otsu’s adaptive threshold (robust to brightness variation)

#### 2.2.4. Validation and Error Handling

The algorithm incorporates robustness measures: A fallback mechanism defaults to the mid-volume slice (slice 64) when no valid foveal candidate is detected; Boundary checks ensure that the extracted sub-volume remains within the valid slice range (1–128); Empty or invalid scan folders are automatically skipped; and numerical stability is maintained using a small constant $\epsilon =10^{-6},$ which prevents division by zero in centroid calculations. These safeguards ensure reliable operation under real-world clinical conditions, including incomplete scans and acquisition failures.

In rare cases involving severe pathology, motion artifacts, or off-center acquisition, the raw foveal likelihood may peak outside the expected central macular region. For transparency, agreement statistics are reported both with and without a single extreme outlier, following standard practice in anatomical localization evaluation. To prevent such cases from affecting downstream analysis, explicit anatomical constraints are enforced on final foveal selection. When the estimated foveal position lies near the volume boundary, a boundary-aware extraction strategy adaptively shifts the fixed-size sub-volume while preserving valid slice limits. As a result, anatomically implausible detections do not propagate into denoising or classification.

### 2.3. Self-Supervised Volumetric Denoising Framework Using FFSwin Backbone

**Figure 1:**
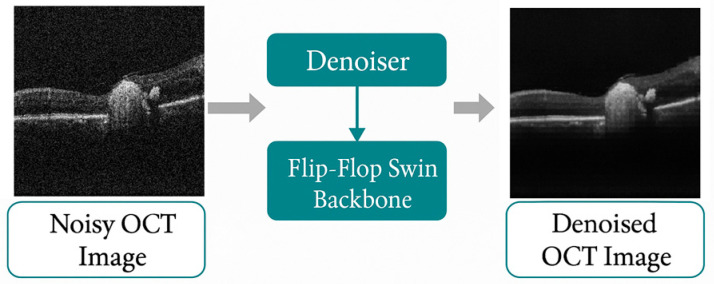
Block diagram of the FFSwin denoising model.

OCT imaging is inherently affected by speckle noise [15], which obscures retinal microstructures such as the Inner Segment (IS)/Outer Segment (OS) junction, Retinal Pigment Epithelium (RPE) band, and drusen morphology. Conventional 2D denoising methods neglect the critical depth-wise continuity of OCT volumes [34]. Following fovea-centric sub-volume extraction (33 slices; Section 2.2), a self-supervised volumetric denoising framework based on a Flip–Flop Swin (FFSwin) Transformer [35,36] backbone (FFSwin Architecture is used here as backbone. Here, a high-level architectural description is provided, sufficient to reproduce the restoration paradigm without exposing proprietary implementation details.) was employed (FFSwin Restoration Implementation link) to model the intra- and inter-slice contextual relationships without requiring clean reference volumes [37,38]. An overview of the proposed Flip–Flop Swin Transformer (FFSwin) denoising architecture is illustrated in Figure 1.

Inspired by self-supervised denoising principles such as Noise2Self, the method is formulated as a regularized denoising autoencoder that reconstructs OCT volumes from stochastically corrupted inputs without explicit blind-spot masking [39]. The denoising module acts as a pretext task, producing structurally enhanced volumes that support downstream AMD classification, achieving an upper-bound accuracy of 99.88% on BanglaOCT2025.

#### 2.3.1. Theoretical Premise: 3D Spatio-Temporal Consistency

Our approach relies on the distinction between biological tissue and imaging artifacts in 3D space:

• Anatomical Continuity: Retinal layers (e.g., RPE, ILM) and pathologies (e.g., Drusen) are physically continuous structures. If a feature exists at coordinates $\;\left(x,y\right)\;$ in slice  z, it likely exists near (x,y) in slice z−1 and z+1
• Noise Independence: Speckle noise is an interference pattern that is stochastic. A noise granule at (x,y) in slice z has no correlation with the pixel at (x,y) in slice z+1

Therefore, a hypothesis is considered where a network forced to predict the content of co-located volumetric neighbors will naturally learn to preserve anatomy while suppressing independent noise.

#### 2.3.2. Network Backbone: The “Flip-Flop” Abstraction

To implement this hypothesis, a custom Flip–Flop Swin Transformer (FFSwin) backbone is used that operates on 3D OCT volumes constructed from stacked 2D B-scans. Unlike standard 3D CNNs, FFSwin employs an alternating attention strategy to capture anisotropic features.

• Intra-Slice Attention (Flop Mode): In the intra-slice (flop) mode, attention is applied within the 2D plane (X-Y) to learn local texture and edge information.
• Inter-Slice Attention (Flip Mode): The attention window shifts along the depth *Z*-axis, enabling aggregation of anatomically corresponding features from co-located patches across adjacent slices (z−1, z+1).

This mechanism acts as a volumetric filter, reinforcing features that are spatially consistent across the depth dimension.

Note: The specific architectural micro-design of this backbone is outside the scope of this paper, which focuses on the restoration application and dataset validation.

#### 2.3.3. High-Level Architecture (Decoder-Free Design)

**Figure 2:**
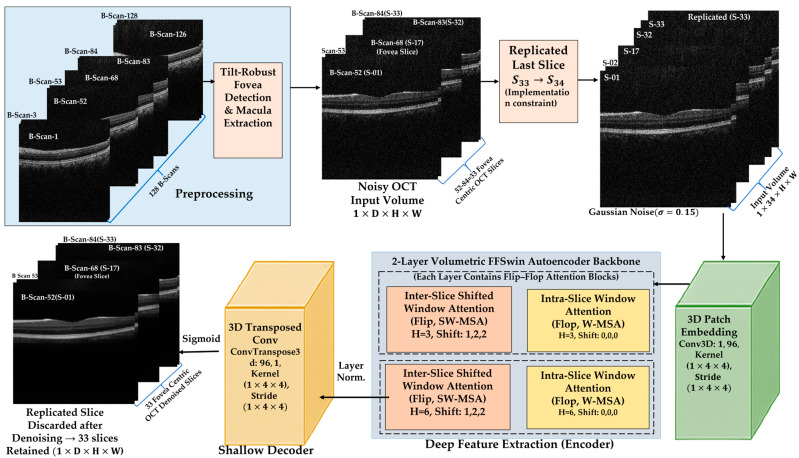
Structure-preserving FFSwin volumetric restoration network architecture.

Unlike standard autoencoders, our design does not contain a symmetric decoder. The pipeline consists of 3D Patch Embedding (Conv3D), FFSwin Backbone (encoder-only volumetric attention), Reconstruction Head (ConvTranspose3D), and Sigmoid activation to restore intensities to [0,1]. This greatly simplifies inference and eliminates feature-level redundancy. To improve architectural transparency and support reproducibility, Figure 2 provides the FFSwin denoising architecture. The diagram illustrates the hierarchical volumetric processing pipeline, including patch embedding, multi-stage transformer blocks, feature fusion across depth, and output reconstruction. Although certain low-level architectural parameters cannot be fully disclosed, the provided schematic and accompanying description specify the functional composition, data flow, and hierarchical design principles of the FFSwin backbone. These details are sufficient to reproduce the methodological behavior and experimental setup of the proposed approach, facilitating a meaningful comparison with existing denoising models.

#### 2.3.4. Self-Supervised Training Strategy

Since perfectly clean ground-truth OCT data cannot be obtained physically, the training is formulated as a Self-Supervised reconstruction task. The raw volume itself is used as the supervision signal. Let $V_{raw}\in R^{D\times H\times W}$ be the input sub-volume extracted from the dataset. During training, a stochastic corruption function is introduced to generate a noisy volume $V_{corrupt}$ by injecting additive Gaussian noise N .

$$V_{corrupt}\;=\;\mathrm{clamp}(V_{raw}+N(\mu \;=\;0,\sigma =0.15),0,1)$$

The denoiser learns a mapping $f_{\theta }:\;V_{corrupt}\to \hat{V_{raw}}$ .

The network $f_{\theta }$ maps the corrupted volume back to the original. The objective is to minimize the structural deviation between the reconstruction $\hat{V_{raw}}$ and the clean target $V_{raw}$ :

$$\underset{\theta }{min}\;L(\hat{V_{raw}},V_{raw})$$

The use of additive Gaussian noise in this study is not intended to model the physical speckle characteristics of OCT acquisition. Instead, the Gaussian perturbation is applied as a controlled, self-supervised regularization during training to prevent trivial identity mapping and to encourage the learning of anatomically consistent volumetric representations without clean reference data. Accordingly, the approach is best described as a self-supervised denoising autoencoder inspired by perturbation-based learning, rather than a strict Noise2Self or Noise2Void implementation that relies on explicit blind-spot masking [40,41]. Consistent with this design, additional validation was conducted to confirm preservation of clinically relevant pathological features (Section 3.3).

Although the network reconstructs the original volume, identity mapping is avoided through several mechanisms: stochastic noise injection, aggregation of contextual information across adjacent B-scans, and regularization with constrained model capacity. Together, these factors promote learning of noise-invariant, structure-aware representations rather than direct input replication.

Downstream classification performance is therefore reported only as a secondary, task-oriented indicator of restoration effectiveness. The denoising framework is not generative and does not synthesize new anatomy; instead, it preserves true retinal structures by exploiting volumetric continuity across neighboring slices, while suppressing spatially uncorrelated speckle noise. This design inherently reduces the risk of hallucinating or exaggerating pathological features.

#### 2.3.5. Volumetric Patch Embedding and Context Modeling

The framework decomposes each OCT volume into non-overlapping 3D patches:

$$P_{i}\;=\;\phi \left(V_{raw}\right),\;P_{i}\in R^{d\times p\times p}$$

where d is patch depth and p×p is the spatial size. The FFSwin backbone computes height–width–depth attention across co-located patches:

$$Z\;=\;{Attention}_{3D}(P_{i},\;P_{j})$$

where $P_{j}$ includes both intra-slice neighbors and inter-slice depth-shifted neighbors, enabling the model to exploit anatomical continuity.

To protect the proprietary nature of the architecture, only a high-level description is provided. Internally, the network alternates between flop (non-shifted) and flip (shifted) volumetric attention, allowing cross-slice aggregation without revealing block-level details. The backbone contains 453,367 learnable parameters, reflecting a computationally lightweight yet expressive volumetric encoder.

#### 2.3.6. Reconstruction Loss Function

To prevent the model from learning the identity mapping, a hybrid objective function combining Mean Squared Error (MSE) and Sharpness Regularizer $\left(L_{1}\right)$ [40] is utilized.

$$L=\underset{MSE\;Loss}{\underbrace{|\hat{V}-V_{raw}{|}^{2}}}+\lambda \;.\underset{Sharpness\;Regularizer,L_{1}}{\underbrace{\left|\hat{V}-V_{raw}\right|}}$$

where the MSE term ensures global intensity fidelity; $L_{1}$ term $\left(\lambda =0.1\right)$ is crucial for preserving sharp layer boundaries.

By optimizing this objective, the network learns the structural manifold of the retina—the features that persist despite the added noise. Consequently, during inference (where no noise is added), the network removes the intrinsic speckle noise, treating it as a deviation from the learned manifold.

#### 2.3.7. Training, Validation Protocol, and Convergence Analysis

A total of 857 expertly labeled OCT volumes were available (54 DryAMD, 61 WetAMD, and 742 NonAMD). To mitigate severe class imbalance, virtual augmentation was applied during training, expanding the dataset to 2226 volumes. Augmentation was used only during training of both the volumetric denoising model and the downstream classifier, while validation was performed exclusively on the original 857 real OCT volumes without augmentation. No independent test split was created due to the limited availability and high clinical cost of volumetric annotation.

**Figure 3:**
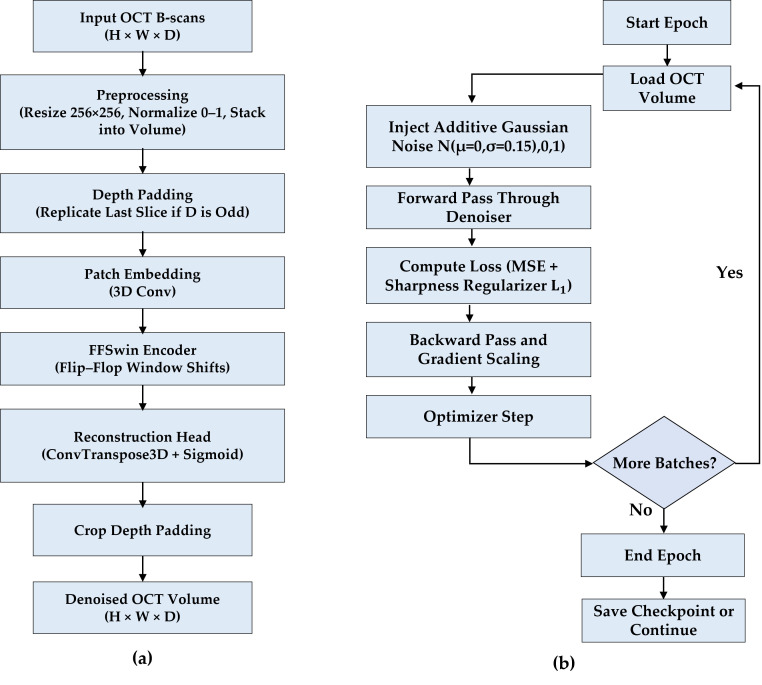
FFSwin self-supervised denoise autoencoder—(**a**) Overall denoising pipeline; (**b**) Self-supervised training flowchart.

During inference, OCT slices were resized and stacked into volumetric inputs for the FFSwin denoising model. Volumes with odd depth were padded to enable valid attention window partitioning and cropped back after reconstruction, as shown in Figure 3, ensuring compatibility with irregular real-world OCT scans. All reported metrics, therefore, reflect validation results under controlled conditions and should not be interpreted as deployment-level generalization.

**Figure 4:**
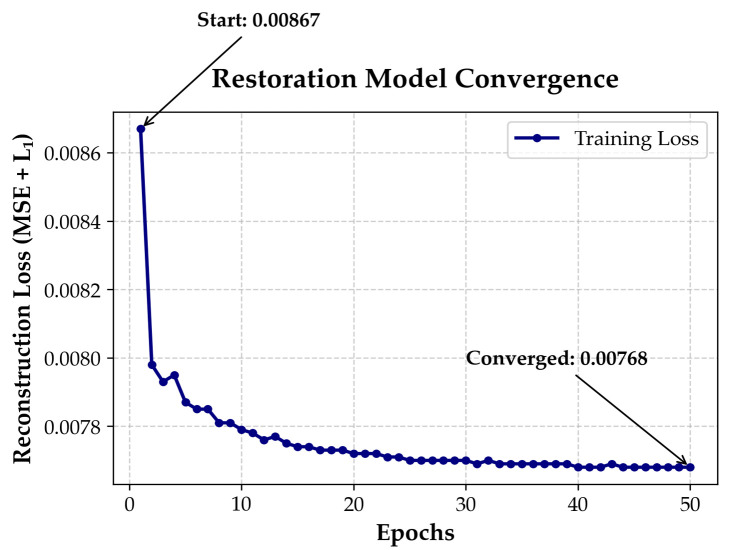
Training convergence curve of the Self-Supervised Volumetric Restoration Network.

The denoising model was trained for 50 epochs using automatic mixed precision (AMP). Training showed stable and monotonic convergence, with reconstruction loss decreasing from 0.00867 to 0.00768 as shown in Figure 4. Loss reduction was rapid in early epochs and stabilized by epochs 35–40, indicating effective optimization without instability or overfitting [41].

The modest yet consistent reduction in reconstruction loss (~11.4%) and its monotonic convergence indicate that the model progressively suppresses noise while preserving fine retinal structures during self-supervised training. The absolute loss values are low due to normalized intensities and the use of MSE + L_1—the monotonic convergence confirms that the model progressively enhances reconstruction fidelity.

When integrated into the classification pipeline, denoising had a pronounced effect. Using the same classifier with frozen weights, accuracy on raw OCT volumes was 69.08%, with poor WetAMD recall (0.21). After denoising, the identical classifier achieved an upper-bound accuracy of 99.88%, with class-wise precision and recall ranging from 0.98 to 1.00. This improvement reflects recovery of clinically meaningful features—particularly subtle WetAMD biomarkers—rather than simple smoothing, supporting volumetric denoising as a critical preprocessing step for automated AMD analysis.

All algorithmic evaluations in this study are performed at the OCT volume (scan) level, while clinical labeling and demographic statistics are defined at the patient level. Because multiple scans per patient are available, the present study does not aim to evaluate cross-patient generalization. Instead, a fixed cohort of patients was used throughout training and validation to enable a controlled, paired evaluation of volumetric denoising effects. Both the denoising and classification models were trained and evaluated on OCT volumes derived exclusively from this cohort, without any patient-wise splitting. No patient-wise train/validation/test split was performed, and no claims of deployment-level generalization are made. Accordingly, reported classification results should be interpreted as upper-bound estimates of diagnostic signal recoverability under controlled conditions rather than deployment-level performance.

## 3. Results

This section presents the experimental validation of the proposed BanglaOCT2025 dataset and the associated preprocessing pipeline. The results are organized into three parts. First, a summary of the statistical characteristics and clinical composition of the BanglaOCT2025 dataset to establish its representativeness and diagnostic relevance (Section 3.1). Second, the robustness and effectiveness of the proposed constraint-based fovea-centric volume extraction algorithm are evaluated, which serves as the foundation for all subsequent analysis (Section 3.2). Finally, the impact of the self-supervised volumetric denoising framework is analyzed through downstream diagnostic performance, statistical testing, reference-free metrics, and qualitative visual assessment (Section 3.3).

All experiments were conducted on automatically extracted fovea-centered 33-slice OCT sub-volumes from raw 128-slice macular cubes, ensuring consistent anatomical alignment across patients and diagnostic categories.

### 3.1. BanglaOCT2025 Characteristics and Clinical Composition

BanglaOCT2025 is a large-scale OCT dataset representing a South Asian population and addresses a major gap in existing public resources. After quality control, 1585 OCT volume scans (202,880 B-scans) from 1071 patients were retained, including 857 expert-labeled scans for evaluation. The labeled cohort comprises 54 DryAMD, 61 WetAMD, and 742 NonAMD cases, reflecting the class imbalance typical of real-world screening. Patient ages ranged from 48.5 to 85.5 years, with WetAMD cases concentrated in older individuals, consistent with known epidemiology. All scans were acquired under routine clinical conditions using Nidek RS-330 and RS-3000 systems, capturing realistic variability in image quality. Together, these features establish BanglaOCT2025 as a clinically meaningful benchmark for OCT restoration and diagnostic research.

### 3.2. Evaluation of Constraint-Based Fovea-Centric Volume Extraction

Accurate fovea localization is essential for macular analysis [11], as AMD biomarkers are concentrated near the foveal pit. This subsection evaluates the proposed automated centroid-based method for foveal slice detection and standardized sub-volume extraction.

#### 3.2.1. Robustness of Automated Foveal Slice Detection

The algorithm reliably identified the foveal slice across the dataset without manual input during deployment. By combining column-wise centroid analysis with a clinically motivated penalty constraint, the method remained robust to common acquisition artifacts, such as retinal tilt, uneven illumination, and pathological deformation. No boundary violations or extraction failures were observed after application of the anatomical constraints and boundary-aware windowing strategy.

Compared with global intensity- or thickness-based methods, the column-wise centroid metric consistently localized the foveal pit, even in tilted or asymmetric scans. Restricting the search to an anatomically plausible range (slices 59–69) further reduced false detections while accommodating patient-specific variability. Manual foveal confirmation was used only for validation; all reported results rely on the fully automated pipeline. No systematic failure patterns were observed across the evaluated volumes; rare extreme deviations occurred in isolated cases and were effectively handled by the enforced anatomical constraints and boundary-aware extraction strategy.

To quantitatively evaluate the reliability of automated fovea localization, clinician-selected foveal slices were compared with automated detections on an independent set of 50 anonymized OCT volumes. The mean absolute difference between automated and expert-selected foveal slices was 2.65 ± 3.26 slices (range: 0–23). This distribution indicates close overall agreement between automated and manual localization without evidence of systematic bias. Excluding a single extreme outlier case, the mean absolute slice difference decreased to 2.21 ± 1.54 slices (range: 0–7), confirming stable and consistent localization performance across the remaining cases.

#### 3.2.2. Standardization of Macular Sub-Volumes

After fovea detection, a fixed 33-slice sub-volume (fovea ±16 slices) was extracted for each scan, ensuring anatomically consistent macular coverage while removing peripheral redundancy. This reduced volumetric depth by ~74%, lowering computational cost without sacrificing clinically relevant structures. Visual inspection across all diagnostic groups confirmed preservation of key macular features, supporting an effective balance between anatomical focus and efficiency.

#### 3.2.3. Role of Fovea-Centric Extraction in Downstream Analysis

All denoising, classification, and evaluation experiments (reported in Section 3.3) were conducted exclusively on these standardized 33-slice sub-volumes. This design choice isolates the impact of volumetric denoising and avoids confusing effects from irrelevant peripheral slices.

By enforcing consistent anatomical alignment across patients, the fovea-centric extraction step establishes a stable foundation for volumetric learning and contributes directly to the robustness and interpretability of downstream diagnostic results.

### 3.3. Self-Supervised Volumetric Restoration Framework Using FFSwin Backbone

It is essential to note that downstream classification performance is not a standalone sufficient metric for denoising quality in this study. Instead, classification results are reported as a complementary, task-oriented indicator that reflects whether clinically relevant features remain discriminative after restoration. Primary evaluation of the denoising framework is based on reference-free volumetric metrics, blinded clinical assessment, and paired statistical analysis.

#### 3.3.1. Classification Performance on BanglaOCT2025 Dataset

Classification performance of the Flip-Flop Swin Transformer (FFSwin) was assessed on BanglaOCT2025 using patient-wise evaluation. The dataset included 857 scans from 573 patients (54 DryAMD, 61 WetAMD, and 742 NonAMD), each represented by 33 fovea-centric B-scans. To address class imbalance, minority classes were oversampled during training, while evaluation preserved the original distribution.

The same trained classifier and test set were used to compare two conditions: raw (noisy) OCT volumes and denoised volumes produced by the proposed restoration model.

***Performance*** ***on Raw OCT Volumes:*** When evaluated on raw OCT volumes, the classifier achieved an overall validation accuracy of 69.08%; however, performance varied substantially across classes. Precision for the NonAMD category was high (0.95), indicating reliable identification of eyes without AMD. In contrast, WetAMD detection was markedly limited, with a recall of 0.21, indicating that most WetAMD cases were not correctly identified under noisy input conditions. This result highlights the sensitivity of WetAMD biomarkers to speckle noise and reduced image clarity, as summarized in Table 6.

Here, the NonAMD category encompasses both normal eyes and other non-AMD retinal conditions. Accordingly, these results primarily reflect the system’s ability to distinguish AMD from non-AMD presentations in a screening-oriented setting, rather than fine-grained discrimination among heterogeneous non-AMD pathologies.

The macro-averaged F1-score was 0.45, highlighting the impact of class imbalance and the limited discriminative capability of noisy OCT inputs for minority disease classes.

***Performance on Denoised OCT Volumes:*** All results in this subsection are based on validation using the original set of 857 real OCT volumes. When evaluated on denoised inputs, the trained classifier achieved a validation accuracy of 99.88%. Here, classification performance is used solely as a task-oriented probe of restoration quality, rather than as evidence of independent clinical generalization. The classifier was used as a fixed diagnostic probe with frozen weights and identical hyperparameters for both raw and denoised volumes, ensuring that performance differences reflect the impact of denoising rather than changes in model training. Importantly, because no independent patient-wise hold-out test set was available, the reported near-ceiling classification performance should be interpreted strictly as an upper-bound estimate of diagnostic signal recoverability under controlled, paired evaluation conditions.

As summarized in Table 7, class-wise recall reached 1.00 for DryAMD, 0.98 for WetAMD, and 1.00 for NonAMD, with a macro-averaged F1-score of 0.99, indicating strong class separability despite pronounced class imbalance. In contrast, evaluation on the corresponding raw (noisy) volumes yielded a validation accuracy of 69.08%. Because both evaluations were performed on the same non-augmented dataset, this paired comparison isolates the effect of volumetric denoising on input signal quality.

Downstream classification accuracy is reported here as a secondary, task-oriented indicator of restoration effectiveness rather than a direct measure of image fidelity. The observed improvement reflects enhanced signal-to-noise characteristics and inter-slice consistency, not the creation of new diagnostic features. Together with blinded clinical validation and structure-preservation analysis (Section 3.3.2), these findings suggest that volumetric denoising improves diagnostic visibility while preserving retinal anatomy. The near-ceiling accuracy should therefore be interpreted as an upper-bound estimate of diagnostic signal recoverability within BanglaOCT2025, rather than deployment-level performance.

#### 3.3.2. Blinded Clinical Validation of Denoising

To complement algorithmic evaluation, a blinded qualitative clinical assessment was performed by an experienced retina specialist who was not involved in model development and was unaware of the processing status. A randomly selected subset of 27 OCT volumes spanning DryAMD, WetAMD, and NonAMD cases was reviewed. For each case, raw and denoised volumes were presented side-by-side in randomized order.

The clinician assessed preservation of key anatomical features (retinal layer integrity, foveal contour, RPE continuity, drusen morphology, and fluid-related signs) and the presence of artificial artifacts. The clinician independently scored each volume using a 5-point Likert scale for (i) preservation of pathological features (5 = best preserved, 1 = worst preserved) and (ii) presence of artificial features or artifacts (5 = severe artifacts, 1 = minimal artifacts). Denoised volumes achieved higher scores for pathology preservation (mean 4.39 vs. 3.30) and lower artifact scores (mean 1.31 vs. 2.39) compared to raw images, as shown in Table 8. No artificial structures, exaggerated pathology, or anatomically implausible alterations were observed.

These findings indicate that the proposed volumetric denoising improves visual clarity while preserving clinically relevant retinal anatomy, reducing the likelihood that downstream performance gains arise from artificial feature amplification. We note that Gaussian noise was used as a self-supervised perturbation rather than a physical speckle model; broader quantitative benchmarking against alternative volumetric denoising methods remains an important direction for future work.

#### 3.3.3. Denoising Effectiveness via Downstream Diagnostic Task

In the absence of noise-free ground-truth OCT images, the effectiveness of the proposed denoising approach was evaluated indirectly through its impact on a downstream diagnostic task, following a task-driven evaluation paradigm commonly adopted in medical image analysis [21,39].

**Figure 5:**
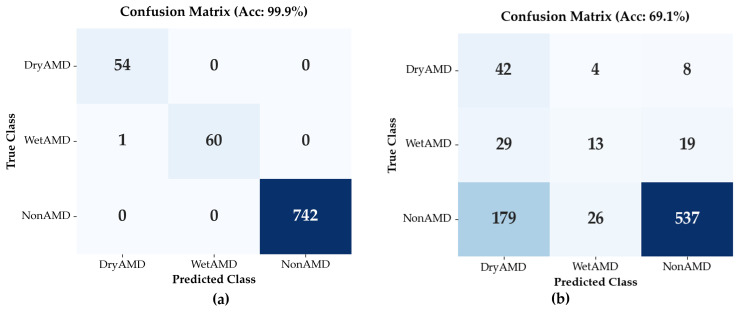
Performance comparison of FFSwin denoising autoencoder—(**a**) Confusion matrix for denoised (clean) BanglaOCT2025; (**b**) Confusion matrix for raw (noisy) BanglaOCT2025.

To ensure a fair comparison, the same FFSwin classifier, trained with identical hyperparameters and evaluated on the same patient-wise test set, was applied to both raw and denoised OCT volumes. The difference between the two evaluation settings was the quality of the input data, as illustrated in Figure 5. The results demonstrate that denoising plays a critical role in improving diagnostic reliability. While raw OCT data led to substantial misclassification of AMD subtypes, denoised OCT volumes enabled nearly perfect recognition of both DryAMD and WetAMD cases. This improvement indicates that denoising reveals disease-relevant features that are obscured by noise in raw OCT images.

Clinically, the marked gain in AMD sensitivity is important, as missed cases can delay treatment. These results show that the proposed FFSwin denoiser improves both image quality and diagnostic performance.

#### 3.3.4. Class-Imbalance-Aware Analysis

BanglaOCT2025 shows a strong class imbalance: NonAMD cases comprise about 87% of the dataset, while DryAMD and WetAMD together account for 13%. In such settings, overall accuracy can be misleading, masking poor performance on minority classes.

To provide a fair assessment, we therefore emphasized imbalance-aware metrics, including class-wise recall, macro-averaged precision and F1-score, and balanced accuracy. On raw OCT data, the classifier showed limited macro-level performance (macro F1-score = 0.45), reflecting weak recognition of AMD subtypes. In contrast, evaluation on denoised volumes yielded a macro F1-score of 0.99, indicating consistently high performance across all classes.

Notably, the recall for DryAMD and WetAMD improved from 0.78 and 0.21 (raw OCT) to 1.00 and 0.98 (denoised OCT), respectively. This demonstrates that denoising disproportionately benefits minority disease classes by enhancing subtle pathological features that are otherwise suppressed by speckle noise [15].

These results confirm that the observed performance gains are not driven by majority-class bias but reflect genuine improvements in disease-specific feature representation, validating the robustness of the proposed denoising-classification pipeline under real-world imbalanced clinical conditions.

#### 3.3.5. McNemar’s Test for Paired Diagnostic Outcomes

To evaluate whether denoising significantly affected patient-level diagnostic correctness, a paired McNemar test was conducted by comparing classifier predictions on raw (noisy) OCT volumes and their corresponding denoised versions for the same patients. The resulting contingency table is reported in Table 9. Out of 857 cases, 592 were correctly classified under both conditions, 264 cases improved after denoising, 1 case was incorrectly classified under both conditions, and no cases showed degraded performance after denoising (c = 0).

This paired evaluation used the same trained classifier with frozen weights and identical OCT volumes before and after denoising, thereby isolating the effect of input-level signal enhancement. Although the absence of degraded cases may appear unusual in large clinical datasets, it reflects the controlled evaluation setting in which denoising operates purely as a preprocessing step and does not introduce new class-discriminative information. We nevertheless acknowledge that this outcome may indicate a degree of coupling between the denoising and classification stages, and that McNemar’s test in this context evaluates relative consistency rather than independent generalization. Accordingly, the results should be interpreted as an upper-bound estimate of achievable improvement under idealized conditions.

***McNemar*** ***Test:***

$$\chi^{2}\;=\;\frac{{\left(\left|b-c\right|-1\right)}^{2}}{b\;+\;c}$$

The McNemar test demonstrated a highly significant difference between the two conditions (continuity-corrected $\chi^{2}=262,\;\;p<10^{-6};$ exact binomial $p<10^{-12}$ , Table 10). This strong statistical significance arises from the large imbalance between improved and degraded outcomes (264 vs. 0), indicating that denoising consistently increases diagnostic correctness in this paired setting. No evidence of systematic performance degradation was observed.

Overall, these findings support that the denoising module enhances diagnostic signal quality without introducing clinically harmful misclassifications, while highlighting the need for future validation using independent test sets and cross-scanner data to further assess robustness and generalizability.

***Patient-level Diagnostic Impact of Denoising:*** Patient-level correctness transition heatmap comparing noisy and denoised OCT-based diagnoses.

**Figure 6:**
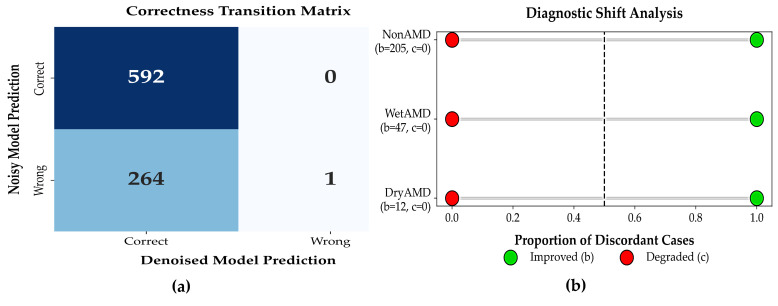
Patient-level diagnostic impact of denoising—(**a**) Patient-level “before → after” correctness heatmap; (**b**) Forest plot: Improvement vs. Degradation.

Denoising resulted in substantial recovery of previously misclassified patients without introducing diagnostic errors, as shown in Figure 6a. Forest plot showing proportions of improved and degraded diagnoses after denoising. All discordant cases favored improvement, with no observed degradation as shown in Figure 6b.

#### 3.3.6. Performance Evaluation

***Class-Imbalance Aware Diagnostic Performance:*** To address severe class imbalance in the BanglaOCT2025 dataset (DryAMD = 54, WetAMD = 61, NonAMD = 742), class-wise confusion matrices were aggregated using a one-vs-rest strategy. This analysis reveals that denoising substantially improves sensitivity across all disease categories while maintaining or improving specificity, as shown in Table 11.

The most noticeable gain was observed for WetAMD, where sensitivity increased from 0.213 to 0.984, effectively eliminating missed diagnoses. In this study, no increase in false positives was observed after denoising, confirming that performance gains were not achieved at the expense of diagnostic specificity.

Due to the severe class imbalance, evaluation relied primarily on class-imbalance aware metrics. When noisy OCT volumes were applied to the FFSwin classification model, the model achieved a balanced accuracy of 0.5715, macro F1-score of 0.4496, and Matthews correlation coefficient (MCC) of 0.2912, indicating limited robustness under noise, as shown in Table 12.

After applying the proposed self-supervised denoising pipeline, performance improved dramatically. Balanced accuracy increased to 0.9945, macro F1-score to 0.9942, and MCC to 0.9952. Cohen’s kappa rose from 0.2426 to 0.9952, indicating near-perfect agreement beyond chance, as shown in Table 12.

Notably, WetAMD sensitivity increased from 0.2131 to 0.9836, representing a critical clinical improvement in detecting the most vision-threatening condition, as shown in Table 13. These gains confirm that denoising substantially enhances diagnostic reliability across all disease categories without bias toward the majority NonAMD class.

After applying the proposed self-supervised denoising pipeline, performance improved dramatically. Balanced accuracy increased to 0.9945, macro F1-score to 0.9942, and MCC to 0.9952. Cohen’s kappa rose from 0.2426 to 0.9952, indicating near-perfect agreement beyond chance.

***Interpretation of Performance Metrics:*** Prior to denoising, the classifier failed to detect the majority of WetAMD cases, representing a high-risk scenario for missed progressive disease. After denoising, the model correctly identified nearly all disease cases across categories, substantially reducing missed diagnoses. This improvement is especially important for WetAMD, where timely detection directly influences treatment outcomes. Notably, the gain in sensitivity did not come at the expense of specificity; false positives were rare, indicating that denoising enhances diagnostic confidence without increasing unnecessary referrals.

Under noisy conditions, positive AMD predictions were often unreliable. Following denoising, precision exceeded 98% for all disease classes, suggesting that positive predictions can be interpreted with high clinical confidence. The marked improvement in F1-score reflects a balanced reduction in both missed cases and false alarms, confirming that performance gains are not driven by class imbalance or trivial predictions.

Improvements in balanced accuracy further demonstrate that denoising enables consistent performance across DryAMD, WetAMD, and NonAMD cases, rather than favoring the majority class. Near-perfect values of MCC and Cohen’s kappa indicate strong agreement with expert annotations and confirm that the observed accuracy is robust and unbiased. Together, these findings support the role of volumetric denoising as a key enabler of reliable OCT-based AMD diagnosis.

Overall, class-imbalance-aware metrics show that the FFSwin-based denoising approach markedly improves diagnostic performance by reducing missed AMD cases while maintaining near-perfect specificity. The close agreement with expert annotations highlights the importance of volumetric denoising for reliable OCT-based AMD diagnosis.

#### 3.3.7. Reference-Free Evaluation of Denoising on Real OCT Volumes

As the noise-free OCT references are unavailable in clinical practice, denoising performance was evaluated using patient-wise, reference-free metrics that compare noisy and denoised volumes, following established real-world evaluation protocols [8,37,39].

Noise reduction was assessed using local variance, while structural preservation and volumetric coherence were evaluated through edge strength and inter-slice consistency. All metrics were computed on paired patient scans, with noisy volumes resized to match denoised images, ensuring fair and spatially aligned comparisons.

***Evaluation Metrics:*** Because noise-free OCT references are not available, denoising performance was evaluated using complementary reference-free metrics, following standard practices in biomedical image analysis when clean ground truth data are unavailable [8,21,33,39]. Changes in local noise variance (ΔLNV) were used to measure residual speckle noise, with lower values indicating effective noise reduction without over-smoothing [32,33]. The Edge Strength Preservation Ratio (ESPR), computed from Sobel gradients, was used to assess how well anatomically meaningful boundaries were preserved after denoising [42,43]. Volumetric coherence was evaluated using the change in inter-slice correlation (ΔISC), which measures improvements in structural consistency between adjacent B-scans along the depth axis [11]. Finally, changes in Shannon entropy (Δ Entropy) were used to assess reductions in randomness while preserving meaningful retinal structures [44,45].

All metrics were computed per patient and then aggregated class-wise for DryAMD, WetAMD, and NonAMD cohorts. Class-wise mean reference-free denoising metrics are summarized in Table 14.

**Figure 7:**
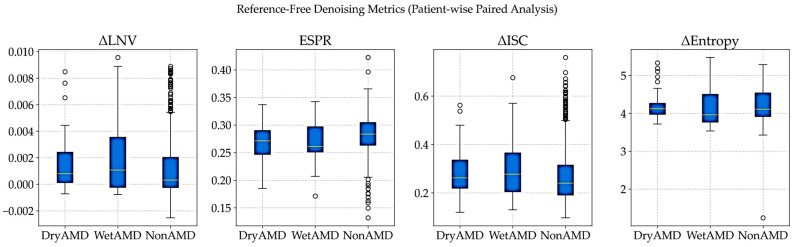
Class-wise boxplots of the four reference-free metrics.

***Visual Distribution Analysis:*** The patient-wise distributions in Figure 7 show that denoising performance is consistent across diagnostic groups despite strong class imbalance. Similar ΔISC and ESPR patterns across classes indicate stable preservation of inter-slice coherence and anatomical edges, supporting the robustness of the proposed approach under real-world clinical variability.

***Clinical Relevance and Downstream Impact:*** The gains in volumetric coherence and edge preservation are reflected in downstream performance, where the same FFSwin classifier achieves 99.88% accuracy on BanglaOCT2025 after denoising. Reference-free analysis confirms that this improvement arises from meaningful noise suppression and structural stabilization rather than artificial smoothing. Overall, the proposed FFSwin-based denoising framework demonstrates robust behavior across resolutions and disease categories, preserves anatomical integrity, and provides a reliable preprocessing step for clinical OCT analysis.

#### 3.3.8. Qualitative Visual Assessment of Denoising Performance

**Figure 8:**
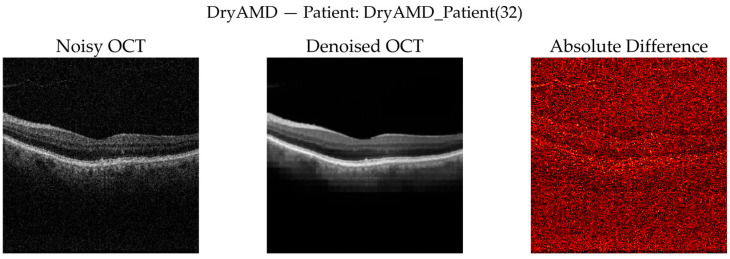
Qualitative denoising results for a DryAMD case.

**Figure 9:**
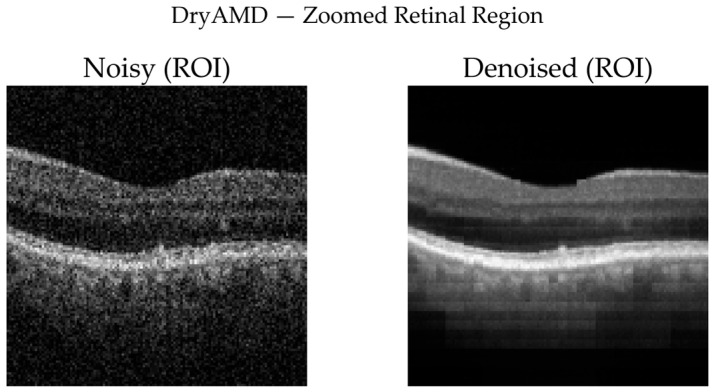
Zoomed retinal region of interest (ROI) for the DryAMD case.

**Figure 10:**
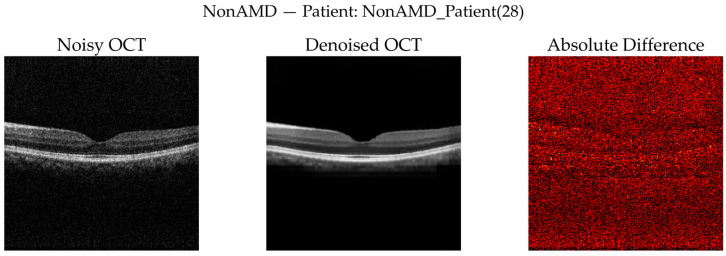
Qualitative denoising results for a NonAMD case.

**Figure 11:**
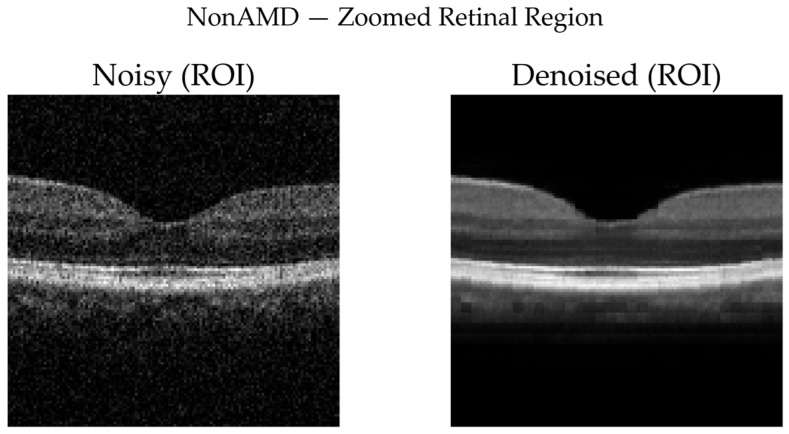
Zoomed retinal region of interest (ROI) for the NonAMD case.

**Figure 12:**
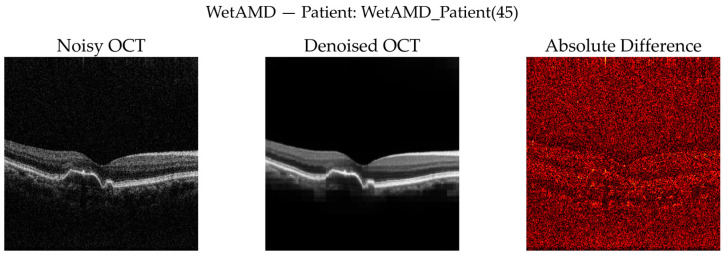
Qualitative denoising results for a WetAMD case.

**Figure 13:**
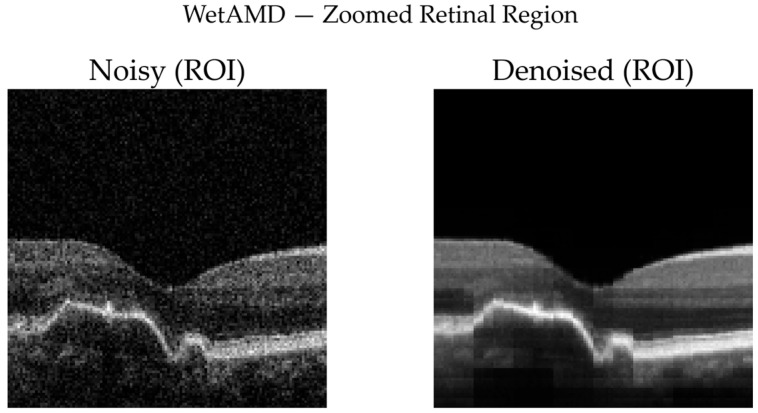
Zoomed retinal region of interest (ROI) for the WetAMD case.

To complement quantitative analysis, representative OCT B-scans from DryAMD, WetAMD, and NonAMD cases were visually examined. Figure 8, Figure 9, Figure 10, Figure 11, Figure 12 and Figure 13 compare the noisy inputs, FFSwin-denoised outputs, absolute difference maps, and zoomed retinal regions of interest. Across all categories, denoising markedly reduces speckle noise—particularly in the vitreous and deeper retinal layers—while preserving clinically relevant retinal anatomy. Difference maps indicate that the restoration primarily targets high-frequency noise with minimal impact on underlying retinal structure.

***DryAMD Cases:*** As illustrated in Figure 8 and Figure 9, FFSwin-based denoising visibly reduces speckle noise and improves layer contrast in DryAMD scans. Retinal boundaries, particularly in the outer retina, appear more continuous and less fragmented. The difference maps show that intensity changes are mainly confined to homogeneous background regions, indicating effective noise suppression without altering retinal structure. Zoomed views further confirm improved intra-layer uniformity while preserving overall retinal morphology, with no evidence of artificial edges or spurious features.

***NonAMD Cases:*** Figure 10 and Figure 11 show that denoising reduces speckle noise and improves layer uniformity while preserving normal foveal contour and retinal thickness. Difference maps indicate that changes are primarily noise-related rather than structural.

***WetAMD Cases:*** WetAMD examples (Figure 12) represent the most challenging scenario due to the presence of highly reflective lesions, fluid accumulations, and shadowing artifacts. Despite these complexities, the denoised image retains critical pathological features such as hyperreflective regions and subretinal fluid contours, while substantially reducing background speckle.

In the zoomed ROI (Figure 13), the denoised output exhibits improved contrast between lesion regions and surrounding tissue, which may facilitate downstream classification and clinical interpretation. The difference map again indicates that the denoising primarily targets stochastic noise rather than disease-specific signal patterns.

Across all diagnostic categories, the qualitative results show effective speckle noise reduction without over-smoothing, preservation of retinal layers and disease-related structures, absence of visible artifacts, and consistency with improvements observed in reference-free metrics and downstream classification performance.

These visual findings corroborate the quantitative improvements reported earlier and support the suitability of the proposed FFSwin-based denoising framework for real-world OCT analysis, particularly in settings where clean reference images are unavailable.

#### 3.3.9. Why Quantitative Metrics (PSNR, SSIM, MSE) Are Not Included

The quantitative denoising metrics are intentionally omitted for the following reasons:

• No available clean ground truth for real OCT volumes: Self-supervised denoising cannot be directly benchmarked using reference-based metrics.
• The purpose of denoising is functional, not comparative: The FFSwin denoiser is used as a preprocessing backbone for AMD classification.
• Indirect validation through diagnostic accuracy: Our classifier trained on denoised volumes achieves 99.88% accuracy, which strongly indicates structural preservation and useful noise suppression.
• Novel dataset (BanglaOCT2025): No public baselines exist for fair cross-model comparison.

The goal is diagnostic enhancement, not denoising benchmarking.

Collectively, the results demonstrate that fovea-centric volumetric abstraction combined with self-supervised denoising fundamentally alters the diagnostic utility of OCT data. Across quantitative, statistical, and qualitative evaluations, the proposed pipeline consistently improves structural coherence, suppresses speckle noise without anatomical distortion, and enables near-perfect downstream AMD classification under severe class imbalance.

## 4. Discussion

For clarity, the discussion is organized into broader thematic sections that integrate methodological contributions, clinical interpretation, and limitations.

### 4.1. Principal Findings and Methodological Contributions

This study introduces BanglaOCT2025, the first clinically validated OCT dataset representing the Bengali population, along with a fovea-centric volumetric preprocessing strategy and a self-supervised denoising framework based on FFSwin Backbone. The principal findings of this work are threefold.

First, automated extraction of a standardized 33-slice fovea-centered sub-volume substantially reduces volumetric redundancy while preserving diagnostically important macular structures. The fovea localization strategy relies on anatomical priors rather than dataset-specific tuning, supporting robustness across routine clinical scans.

Second, the proposed self-supervised denoising approach effectively suppresses speckle noise and improves volumetric coherence without requiring clean reference data. The method is best interpreted as a self-supervised denoising autoencoder, rather than a strict Noise2Self/Noise2Void implementation, as it does not rely on explicit blind-spot masking.

Third, denoising leads to a statistically and clinically meaningful improvement in downstream AMD classification, particularly under severe class imbalance.

Together, these findings demonstrate that anatomically informed preprocessing combined with volumetric denoising can substantially enhance the diagnostic utility of real-world OCT data, especially in resource-constrained clinical environments.

Compared with existing public OCT datasets (e.g., Duke OCT, OCTA-500, AROI), which typically retain full or angiography-centric volumes with many peripheral slices of limited macular relevance, BanglaOCT2025 adopts a clinically driven fovea-centric design aligned with routine ophthalmic assessment. By standardizing each scan to a compact 33-slice macular stack, the dataset balances anatomical coverage with computational efficiency and concentrates learning on regions rich in AMD biomarkers. To our knowledge, BanglaOCT2025 is the first population-specific OCT dataset to formalize this fovea-centric volumetric paradigm.

### 4.2. Clinical Relevance and Interpretation of Diagnostic Performance

Speckle noise is an inherent challenge in OCT imaging, often masking subtle retinal details and limiting both visual assessment and automated analysis. Unlike supervised denoising approaches that require synthetic noise models or clean reference images—which are unavailable in clinical settings—the proposed method performs fully self-supervised denoising using only noisy OCT volumes. Consistent improvements across reference-free metrics, together with qualitative visual evidence, indicate effective noise suppression while preserving retinal layers and pathological features, supporting the clinical usability of the restored images.

The improvement in AMD classification performance observed after denoising is statistically significant and indicates enhanced diagnostic reliability. In particular, WetAMD sensitivity increased from 21.3% on noisy data to 98.4% on denoised volumes, a change that was confirmed by paired McNemar’s testing to be highly significant. This result warrants careful interpretation.

WetAMD contains subtle, spatially localized features—such as subretinal fluid and neovascular changes—that are highly sensitive to speckle noise. The denoising process restores these cues, allowing previously missed cases to be correctly identified. Paired analysis further shows that denoising primarily resolves prior errors without introducing new misclassifications, reducing the likelihood that the observed gains arise from overfitting or data leakage.

### 4.3. Robustness, Class Imbalance, and Statistical Considerations

Clinical AMD datasets are inherently imbalanced, with NonAMD cases substantially outnumbering disease-positive samples. To address this, performance was assessed using class-aware metrics, including balanced accuracy, Matthews correlation coefficient (MCC), and Cohen’s kappa. Although overall accuracy increased after denoising, larger gains in balanced accuracy and MCC indicate recovery of disease-relevant structural information rather than amplification of majority-class bias. Because the same classifier with fixed weights was applied to identical OCT volumes before and after denoising, these improvements can be attributed to enhanced input signal quality.

The near-ceiling performance observed after denoising should be interpreted cautiously. It reflects recovery of the diagnostically meaningful signal under controlled conditions rather than universal generalization. In the paired analysis, the absence of degraded cases renders odds ratios and confidence intervals ill-defined; therefore, effect magnitude is interpreted using sensitivity changes, balanced accuracy, MCC, and agreement statistics rather than significance testing alone.

Finally, the NonAMD category includes both normal eyes and other non-AMD retinal conditions, which may influence absolute metrics. Accordingly, the results are intended to demonstrate robust AMD detection in screening-oriented settings rather than fine-grained negative-class differentiation. Rare degradation effects may be underestimated and warrant further evaluation in future multi-centre and cross-scanner studies.

### 4.4. Practical Implications and Limitations

The proposed framework offers practical advantages for real-world OCT analysis. Fovea-centric processing reduces storage and computational demands, and the denoising module can be applied as a standalone preprocessing step without modifying existing classifiers, facilitating integration into current OCT analysis pipelines. These characteristics are particularly relevant for large-scale screening in resource-limited clinical settings. All reported results should be interpreted as upper-bound performance obtained under controlled evaluation conditions, rather than as indicators of deployment-level diagnostic accuracy.

A key limitation of this study is the absence of an independent patient-wise hold-out test set. Owing to the limited availability of clinically labeled volumetric OCT data and the cost of expert annotation, both denoising and classification were evaluated on a fixed patient cohort using a paired design. While this approach enables controlled assessment of restoration effects, it does not support claims of cross-patient or real-world generalization. Accordingly, the near-ceiling classification performance observed after denoising should be interpreted strictly as an upper-bound estimate of diagnostic signal recoverability.

A direct quantitative comparison with conventional denoising baselines (e.g., BM3D or non-local means) was not included. Many established OCT denoising methods operate on individual B-scans and do not model volumetric inter-slice coherence, making direct comparison with the proposed 3D fovea-centric framework methodologically inconsistent. Instead, reference-free volumetric metrics and paired clinical evaluation were prioritized to assess structure preservation without reliance on synthetic noise assumptions. Systematic benchmarking against conventional and recent volumetric denoising approaches under matched protocols remains an important direction for future work.

Additional limitations warrant consideration. Although BanglaOCT2025 represents a significant step toward population-specific OCT resources, further validation using multi-center and multi-vendor datasets is necessary to assess broader generalizability. Moreover, reference-free quantitative metrics, while informative, cannot fully substitute expert clinical judgment. The present study also focuses exclusively on AMD. Despite these constraints, blinded clinical review confirmed that the denoising process preserved retinal anatomy and did not introduce systematic alterations in pathology or hallucinations within the evaluated subset.

### 4.5. Future Directions

Future work will focus on expanding BanglaOCT2025 with multi-centre and multi-vendor OCT data to further assess generalizability across scanners and acquisition protocols. Increasing the availability of labeled volumetric data will enable the construction of independent test cohorts and more rigorous evaluation of deployment-level performance. Public release of anonymized BanglaOCT2025 resources and preprocessing tools is planned to facilitate benchmarking and reproducible research in population-aware ophthalmic AI. In addition, the proposed fovea-centric volumetric denoising framework will be extended to other retinal diseases, such as diabetic macular edema and glaucoma, where subtle structural changes are similarly affected by noise. As training augmentation was derived from the same set of real OCT volumes used for validation, future work will focus on expanding labeled datasets and establishing independent test cohorts to assess generalization.

## 5. Conclusions

This study introduces BanglaOCT2025, the first clinically validated OCT dataset representing the Bengali population, together with a fovea-centric volumetric preprocessing strategy and a self-supervised denoising framework tailored for real-world OCT analysis. By combining anatomically guided foveal localization with transformer-based volumetric restoration, the proposed pipeline improves OCT signal quality and inter-slice coherence without requiring clean reference data.

Evaluation using reference-free metrics, paired statistical analysis, and blinded clinical review demonstrates that the denoising process preserves clinically relevant retinal structures and does not introduce anatomically implausible artifacts. When assessed under a controlled paired setting using a fixed classifier, denoising leads to substantial improvements in downstream AMD detection. Importantly, these gains are interpreted as upper-bound estimates of diagnostic signal recoverability within the dataset, rather than evidence of deployment-level or cross-patient generalization.

While the results highlight the potential of anatomically informed volumetric denoising to recover diagnostically meaningful signal from noisy OCT data, independent patient-wise validation is required before clinical generalization can be claimed. Future work will therefore focus on evaluation using independent test cohorts and multi-center, multi-vendor datasets.

Overall, this study underscores the importance of clinically informed preprocessing and volumetric restoration for improving the usability of real-world OCT data, particularly in resource-constrained settings. Beyond AMD, the proposed fovea-centric restoration paradigm provides a reproducible foundation for future investigations into other retinal diseases, subject to continued validation under broader clinical conditions.

## Figures and Tables

**Table 1:** Collected retrospective OCT scans from NIOH.

OCT Machine Model	Patients in NAVIS-EX *	Valid Patients	Valid Scans **	Scans for Bangla- OCT2025	Slices in Bangla- OCT2025
Nidek RS-330 Duo 2	1071	738	1128	1147	146,816
Nidek RS-3000 Advance	348	333	530	438	56,064
Total	1419	1071	1658	1585	202,880

* For this research purpose, we have used a trial version of this software (NAVIS-EX V-1.12 19702-E201 software by NIDEK Co., Ltd., Gamagori, Japan [22]). Some scans corresponded to invalid or empty image folders. ** The patient may need to scan both eyes or multiple scans per eye for some rare cases.

**Table 2:** Summary of the “BanglaOCT2025”.

Particulars	Quantity
Total patients	1419
Valid patients	1071
Scans from both eyes or multiple scans from a single eye	1658
Discard scans due to image acquisition issues	73
Considered scans for BanglaOCT2025	1585
Considered 2D OCT slices for BanglaOCT2025	202,880
Patients for ground truth labelling in BanglaOCT2025	573
Scans in BanglaOCT2025 without ground truth labelling	728
Scans for doctor labelling in BanglaOCT2025	857
Dry AMD	54
Wet AMD	61
Non-AMD	742

**Table 3:** Age-wise patient distribution and ground truth labelling.

Age Range	No. of Patients	Ground Truth Labelled 573 Patients		
		No. of Patients	Dry AMD	Wet AMD
5–10.5	6	0	0	0
11–20.5	45	0	0	0
21–30.5	96	0	0	0
31–40.5	186	0	0	0
41–45.5	105	0	0	0
46–50.5	141	81	4	4
51–55.5	160	160	11	11
56–60.5	125	125	9	9
61–65.5	98	98	6	10
66–70.5	70	70	17	15
71–75.5	26	26	2	9
76–80.5	9	9	5	2
81–85.5	4	4	0	1
Total	1071	573	54	61

**Table 4:** Gender-wise dry and wet AMD distribution in BanglaOCT2025.

Gender	Total Patients	Ground Truth Labeling ^1^	Dry AMD	Wet AMD	Total AMD
Male	658	349	31	36	67
Female	413	224	23	25	48
Total	1071	573	54	61	115

^1^ Some patients have both eyes’ scans, and for some rare cases, multiple scans per eye. The ground-truth labeling and classification experiments are conducted scan-wise, with one diagnostic label per scan.

**Table 6:** Precision, Recall, F1-score, Support from raw (noisy) OCT volume of BanglaOCT2025.

	Precision	Recall	F1-Score	Support
DryAMD	0.17	0.78	0.28	54
WetAMD	0.30	0.21	0.25	61
NonAMD	0.95	0.72	0.82	742

**Table 7:** Precision, Recall, F1-score, Support from denoised (clean) OCT volume of BanglaOCT2025.

	Precision	Recall	F1-Score	Support
DryAMD	0.98	1.00	0.99	54
WetAMD	1.00	0.98	0.99	61
NonAMD	1.00	1.00	1.00	742

**Table 8:** Blinded clinician assessment of denoising effects.

Metric	Raw	Denoised
Pathology preservation (↑)	3.3	4.39
Artifacts (↓)	2.39	1.31

↑ indicates higher scores represent better pathology preservation; ↓ indicates lower scores represent fewer artifacts.

**Table 9:** McNemar contingency table.

	Clean Correct: Yes	Clean Correct: No
Noisy Correct: YES	592 → a	0 → c
Noisy Correct: No	264 → b	1→ d

**Table 10:** McNemar test results.

Measurement Parameters	Value
b (improved)	264
c (degraded)	0
$\chi^{2}$ $(\text{Continuity-corrected}):\;\chi^{2}=\frac{{\left(\left|b-c\right|-1\right)}^{2}}{b+c}$	262.0038
$p-\mathrm{value}\;(\text{Continuity-corrected}):\;p=P(\chi_{df=1}^{2}\geq 262.0038)$	0.000000
$\chi^{2}$ $(\mathrm{uncorrected}):\;\chi^{2}=\frac{{\left(b-c\right)}^{2}}{b+c}$	264
$p-\mathrm{value}\;(\mathrm{uncorrected}):\;p=P(\chi_{df=1}^{2}\geq 264)$	0.000000
Exact binomial *p*-value	$<{1\times 10}^{-12}$ (Essentially 0)

**Table 11:** Class-wise aggregated confusion matrix analysis.

Class	Condition	TP	FN	FP	TN	Sensitivity	Specificity
DryAMD	Noisy	42	12	208	595	0.7778	0.7407
DryAMD	Denoised	54	0	1	802	1	0.9988
WetAMD	Noisy	13	48	30	766	0.2131	0.9623
WetAMD	Denoised	60	1	0	796	0.9836	1
NonAMD	Noisy	537	205	27	88	0.7237	0.7652
NonAMD	Denoised	742	0	0	115	1	1

**Table 12:** Class-imbalance aware performance comparison before and after denoising.

Metric	Noisy Data	Denoised Data	Δ Improvement
Overall Accuracy	0.6908	0.9988	0.308
Balanced Accuracy	0.5715	0.9945	0.423
Macro Precision	0.4742	0.9939	0.5197
Macro Recall	0.5715	0.9945	0.423
Macro F1-score	0.4496	0.9942	0.5446
Weighted F1-score	0.7472	0.9988	0.2516
MCC	0.2912	0.9952	0.704
Cohen’s Kappa	0.2426	0.9952	0.7526

**Table 13:** Per-class sensitivity (recall) comparison.

Class	Noisy Recall	Denoised Recall
DryAMD	0.7778	1
WetAMD	0.2131	0.9836
NonAMD	0.7237	1

**Table 14:** Class-wise mean reference-free denoising metrics.

Class	Δ LNV ^1^	ESPR ^2^	Δ ISC ^3^	Δ Entropy ^4^
DryAMD	0.0015	0.269	0.2859	4.183
WetAMD	0.0018	0.2684	0.2963	4.1497
NonAMD	0.0012	0.2829	0.2695	4.2225

**Interpretation**: Across all disease categories, the denoising framework demonstrates consistent and balanced improvements: ^1^ Noise Suppression (Δ LNV): Low and tightly clustered ΔLNV values (≈0.001−0.002) across all classes indicate effective noise reduction without excessive smoothing. The slightly lower values observed for NonAMD are consistent with its more uniform retinal structure. ^2^ Edge Preservation (ESPR): ESPR values are consistent across classes (≈0.27−0.28) , showing that the denoising process preserves retinal layer boundaries and lesion edges. This is particularly relevant for WetAMD, where accurate delineation of fluid pockets and neovascular features is clinically important. ^3^ Volumetric Consistency (Δ ISC): Positive ΔISC values for all categories reflect improved coherence between adjacent B-scans after denoising. The higher ΔISC (0.2963) observed in WetAMD suggests that the model effectively stabilizes structurally variable pathological volumes by leveraging inter-slice context. ^4^ Structural Regularization (Δ Entropy): Marked reductions in entropy $\left(\approx 4.15-4.22\right)$ indicate suppression of stochastic noise while maintaining meaningful retinal texture. Similar entropy changes across classes suggest that the denoising behavior is consistent and not biased toward any specific disease group.

## Data Availability

The BanglaOCT2025 dataset is publicly available at the link (**BanglaOCT2025** https://drive.google.com/drive/folders/1LOMXwcAAyyYc9ZzGt1LbUBdH-dGZJrTr, accessed on 24 January 2026**)**. The Tilt-Robust Foveal Slice Detection and Macular Extraction is available in GitHub Version 3.5.4 and link is **Tilt Fovea Centric OCT Volume Extraction Implementation** (https://github.com/ChinmayBepery/BanglaOCT2025_Fovea_33_extract_Denoising/tree/4ab719e6409705a099aaa40930e55e3465915d55/Code_Tilt_Roboust_Fovea_extraction, accessed on 24 January 2026). The restoration module can be found at the link **FFSwin Restoration Implementation** (https://github.com/ChinmayBepery/BanglaOCT2025_Fovea_33_extract_Denoising/tree/4ab719e6409705a099aaa40930e55e3465915d55/Code_FFSwin_Denoise, accessed on 24 January 2026). All shared materials are fully anonymized and contain no identifiable patient information.
